# Palmitoylation Regulates Intracellular Trafficking of β_2_ Adrenergic Receptor/Arrestin/Phosphodiesterase 4D Complexes in Cardiomyocytes

**DOI:** 10.1371/journal.pone.0042658

**Published:** 2012-08-09

**Authors:** Ruijie Liu, Dayong Wang, Qian Shi, Qin Fu, Steven Hizon, Yang K. Xiang

**Affiliations:** 1 Department of Molecular and Integrative Physiology, University of Illinois at Urbana Champaign, Urbana, Illinois, United States of America; 2 Department of Pharmacology, University of California Davis, Davis, California, United States of America; University of Texas Health Science Center at Houston, United States of America

## Abstract

β_2_ adrenergic receptor (β_2_AR) is a prototypical G-protein coupled receptor that stimulates the classic cAMP-protein kinase A (PKA) signaling pathway. Recent studies indicate that the cAMP-PKA activities are spatiotemporally regulated in part due to dynamic association of β_2_AR with phosphodiesterase 4D (PDE4D), a group of cAMP degradation enzymes. Here, we demonstrate that in cardiomyocytes, palmitoylation of β_2_AR, the covalent acylation of cysteine residue 341, plays a critical role in shaping subcellular cAMP-PKA activities in cardiomyocytes via regulating β_2_AR association with arrestin/PDE4D. Replacing cysteine 341 on β_2_AR with alanine (C341A) leads to an impaired binding to β arrestin 2. Surprisingly, the C341A mutant is able to internalize via an arrestin-independent pathway at saturated concentration of agonist stimulation; the internalization becomes caveolae-dependent and requires dynamin GTPase. However, the impaired binding to β arrestin 2 also leads to an impaired recruitment of PDE4D to the C341A mutant. Thus, the mutant C341A β_2_AR is transported alone from the plasma membrane to the endosome without recruiting PDE4D. This alteration leads to an enhanced cytoplasmic cAMP signal for PKA activation under β_2_AR stimulation. Functionally, Mutation of the C341 residue or inhibition of palmitoylation modification of β_2_AR enhances the receptor-induced PKA activities in the cytoplasm and increases in myocyte contraction rate. Our data reveal a novel function of palmitoylation in shaping subcellular cAMP-PKA signaling in cardiomyocytes via modulating the recruitment of β arrestin 2-PDE4D complexes to the agonist-stimulated β_2_AR.

## Introduction

β_2_AR plays important roles in cardiovascular and pulmonary physiology [1], [2]. Upon activation by catecholamines, β_2_AR couples to stimulatory G protein, and activates adenylate cyclase (AC) to produce cAMP. cAMP-dependent PKA phosphorylates various proteins in cardiomyocytes, such as phospholamban, L-type calcium channel, and troponins, resulting in increased heart contraction force and rate [3].

Recently, PDE4D enzymes have been shown to control basal levels of receptor phosphorylation by PKA via binding to β_1_AR and β_2_AR and controlling local cAMP levels [4], [5]. Specifically, β_1_AR selectively binds to PDE4D8 [6] whereas β_2_AR binds broadly to different PDE4D isoforms with strongest binding to PDE4D9 at basal condition [7]. Moreover, agonist stimulation leads to β_2_AR association with PDE4D5 and PDE4D8 specifically. The recruitment of PDE4D isoforms plays a critical role in controlling the spatiotemporal distribution of cAMP signal for selective activation of PKA, and subsequent phosphorylation of substrates for contractile responses [7].

Over the last decade, several types of post-translational modifications on β_2_AR have been characterized. They are implicated in receptor structure and signaling in mammalian cells [8]–[13]. Among them, the role of β_2_AR phosphorylation by G protein-coupled receptor kinases (GRKs) has been implicated in receptor association with PDE4D enzymes. Upon agonist stimulation, GRK-mediated β_2_AR phosphorylation leads to recruitment of β arrestin 2, which serves as a scaffold protein to recruit PDE4D5 and PDE4D8 [7]. The increased binding of PDE4D isoforms to the activated receptor plays an essential role in confining the cAMP-PKA activities in local environments and dictating the specificity of receptor signaling. In comparison, the role of β_2_AR palmitoylation and its effects on receptor signaling and function in cardiomyocytes remain to be addressed.

Palmitoylation can be enhanced by isoproterenol stimulation [14], [15]. Losing β_2_AR palmitoylation leads to higher basal cAMP-PKA activity. Early studies suggest that the palmitoylation of β_2_AR reduces the receptor coupling to Gs proteins for activation of adenylate cyclase and PKA phosphorylation of receptor in Chinese hamster fibroblasts [16], the functional implication remains unclear. In this context, we hypothesize that palmitoylation of β_2_AR affects the receptor signaling and function in cardiomyocytes.

Thus, we set out to study how β_2_AR palmitoylation influences receptor signaling pathway in cardiomyocytes. Specifically, we like to examine whether palmitoylation of β_2_AR affects agonist-induced receptor trafficking and receptor association with other effectors like PDE4D isoforms in signaling complex. Here, we validate that mutation of β_2_AR at palmitoylation site C341A leads to less cAMP production on the plasma membrane, consistent with the impaired Gs/AC signaling as previously reported. More interestingly, we find that the mutant receptor displays reduced association with PDE4Ds, due to that it has reduced recruitment of β arrestin-2. Surprisingly, the mutant receptor is able to internalize via an arrestin-independent pathway. Therefore, the mutant receptor internalizes alone without recruitment of PDE4Ds, leading to altered spatial distribution of cAMP signal in cardiomyocytes, low cAMP signal at the plasma membrane, and higher cAMP signal in the cytoplasm. Functionally, inhibition of β_2_AR palmitoylation or mutation of cysteine 341 leads to higher PKA activity in the cytoplasm and increase in myocyte contraction rate. In conclusion, our data indicate that palmitoylation of β_2_AR is required for receptor association with arrestin-PDE4Ds to shape subcellular cAMP level in cardiomyocytes for contraction response.

## Materials and Methods

### Site Mutagenesis, Adenovirus Preparation and Cell Culture

Mouse β_2_AR palmitoylation mutant (flag-β_2_AR-C341A) was generated through site directed mutagenesis of cysteine 341 of β_2_AR to alanine using pCDNA3.1-flag-β_2_AR as DNA template. The mutant was sequenced and subcloned into adenovirus vector pAdEasy-1 through recombination in bacteria BJ5831 for adenovirus production in HEK293-QBI cells. Adenoviruses expressing, N terminal PDE4D5-GFP, PDE4D8-GFP, PDE4D9-GFP, and full length PDE4D5-RFP, PDE4D9-HA were created as described previously [7]. Adenoviruses expressing adenylate cyclase isoform VI (AC-VI) had been reported previously [17]. AKAP79-GFP was a gift from Dr. John Scott (University of Washington). Dominant negative β arrestin 2-V54D and dynamin K44E were described elsewhere [18]. HEK293 cells and mouse embryonic fibroblasts [7] (MEFs, a gift from Dr. Robert Lefkowitz, Duke University) lacking arrestin genes were cultured in Dulbecco’s Modified Eagle’s Medium (DMEM) media before transfected with plasmids expressing receptors and other constructs as indicated. Cells were stimulated before being processed for immunofluorescence imaging.

### Neonatal Cardiomyocytes Isolation, Total Intracellular cAMP Measurement, and Myocyte Contraction Rate Measurement

β_1_AR and β_2_AR double knockout (β_1_β_2_AR-KO) cardiomyocytes were isolated by digestion of neonatal mouse heart tissue with type II collagenase (Worthington, NJ) and cultured in DMEM supplied with 10% fetal bovine serum. After overnight culture, cells were washed with phosphate buffered saline (PBS) to remove dead cells and then infected with adenoviruses expressing β_2_AR, β_2_AR-C341A for 24 hours. For total intracellular cAMP measurement, cells were treated in the presence or absence of 10 µM isoproterenol for 5 minutes or β_2_AR selective inverse agonist ICI118551 (1 µM, Tocris, MN) for 30 minutes. Cells were treated with 2-bromopalmitic acid (100 µM, Sigma, MO) 15 minutes to inhibit palmitoylation of β_2_AR, which has half-life of 9.8 minutes [15]. Total intracellular cAMP level was measured using cAMP HTS immunoassay kit (Millipore, MA). Detailed procedure for measuring myocyte contraction rate has been described previously [19].

### Radioligand Binding Assay

Neonatal cardiomyocytes overexpressing flag-β_2_AR or flag-β_2_AR-C341A were washed once with cold PBS, harvested into hypotonic lysis buffer (20 mM HEPES, pH 7.4, 1 mM EDTA, 1 mM PMSF, and 1 mM benzamidine). Cells were homogenized for 20 strokes in a tight homogenizer followed by centrifugation for 10 minutes at 800 g to remove nuclei and unbroken cells. The supernatant was further spun down for 20 minutes at 18,000 rpm using a Beckman JC-M2 rotor to pellet the membrane, which was resuspended by passage through 25-gauge needle in 1× binding buffer (75 mM Tris-HCl, pH 7.4, 12.5 mM MgCl_2_, 1 mM EDTA). After measuring protein concentration by BCA assay (Pierce, IL), 20 µg of membrane protein containing β_2_AR was incubated with 10 nM [^3^H] dihydroalprenolol (GE Healthcare, Piscataway, NJ) for 1 hour at shaken speed of 230 rpm under room temperature. 20 µg of membrane proteins from cardiac cells without overexpression of β_2_AR were used as control. Membranes were harvested and rinsed with 1× binding buffer. The membrane-bound [^3^H] dihydroalprenolol was detected using a Beckman LS 6500 liquid scintillation counter (Beckman Coulter, Fullerton, CA).

### Receptor Internalization and Recycling

β_1_β_2_AR-KO cardiomyocytes were infected with adenoviruses expressing flag-β_2_AR, flag-β_2_AR-C341A for 24 hours. Cells were serum starved for 30 minutes, and in some cells, treated with 2 µg/ml filipin for 30 minutes, and then stimulated with 10 µM isoproterenol for different time as indicated to examine receptor internalization. For determination of receptor recycling, cells were stimulated with 10 µM isoproterenol for 10 minutes followed by drug removal to allow receptor recycling for 30 minutes and 60 minutes. Cells were fixed with 4% paraformaldehyde and permeabilized with 0.2% Nonidet P-40 and stained with anti-flag antibody (mouse IgG_2b_, Sigma, MO), followed by Alex-594 conjugated secondary antibody against mouse IgG_2b_ (Invitrogen, CA). Images were captured with a CCD camera on a Zeiss microscope with MetaMorph software. Quantification of receptor internalization and recycling using FLISA method had been described in previous studies [20].

### Purification of Caveolin-rich Membrane Fractions

Neonatal cardiomyocytes expressing flag-β_2_AR or flag-β_2_AR-C341A were collected into cold PBS and harvested by centrifugation. Cells were resuspended in 2 ml 0.5 M Na_2_CO_3_ (pH∼11). After gentle vortexing, 1.75 ml sample was mixed with 1.75 ml 80% sucrose buffer (25 mM MES, pH 6.5, 0.15 M NaCl, 80% sucrose) and loaded onto the bottom of ultracentrifuge tube. A two-step gradient was loaded onto the top of the sample with 3.5 ml 35% sucrose buffer (25 mM MES, 0.15 M NaCl, 250 mM Na_2_CO_3_, 35% sucrose) and 4 ml 5% sucrose buffer (25 mM MES, 0.15 M NaCl, 250 mM Na_2_CO_3_, 5% sucrose). Sample was spun down in a Beckman SW41 rotor for 18 hours at 38,000 rpm. 1 ml fraction was collected from top to bottom and mixed with 10% trichloroacetic acid for protein precipitation for 4 hours on ice. The protein pellet was dissolved in 1× SDS-sample buffer. Proteins after precipitation was collected by centrifugation for 30 minutes at 13,200 rpm, washed twice with 100% cold acetone and dissolved in 1× SDS-sample buffer. Proteins were separated by 12% SDS-PAGE for detection of caveolin-3 (BD, NJ), flag-β_2_AR or flag-β_2_AR-C341A by anti-flag antibody (Sigma, MO), and Src (Cell Signaling, MA).

### Co-immunoprecipitation and Western Blot

Cells overexpressing flag-β_2_AR, flag-β_2_AR-C341A, or together with individual PDE4D isoforms or GFP-β arrestin 2 were stimulated with 10 µM isoproterenol and harvested into lysis buffer (20 mM HEPES, pH 7.4, 0.6% Nonidet P-40, 150 mM NaCl, 2 mM EDTA, 10% glycerol, 0.03% n-Dodecyl-β-D-maltoside (Enzo Life Sciences, PA), 1 mM PMSF, 1 mM benzamidine, 1 mM NaF, 1 mM Na_3_VO_4_). Protein samples were lysed for 40 minutes at 4°C before centrifugation for 10 minutes at 16,000 g to collect supernatant. Receptors were immunoprecipitated by anti-flag M2 affinity resin (Sigma, MO) for 2 hours at 4°C and eluted into 2x SDS-sample buffer for SDS-PAGE. After transfer onto nitrocellulose membrane, proteins were blotted with 5% non-fat milk in 1x TBST and subsequently blotted with the following antibodies: endogenous PDE4 (Abcam, CA), GFP (Clontech, CA), anti-flag (Sigma, MO), caveolin-3 (BD, NJ), RFP (Thermofisher, IL), HA (Covance, CA). The optical density of the bands was analyzed with ImageJ (National Institute of Health, MD, USA).

### Receptor Phosphorylation

Cardiomyocytes expressing flag-β_2_AR or flag-β_2_AR-C341A were serum starved for 30 minutes before stimulation with 10 µM isoproterenol for different time as indicated. Proteins were harvested and subjected to SDS-PAGE for Western blot with antibodies against total β_2_AR, phospho-β_2_AR by PKA on serines 261/262 [21], and phospho-β_2_AR by GRK on serines 355/356 (SCBT, CA).

### Fluorescence Resonance Energy Transfer (FRET)

The detailed procedures to measure real time cAMP/PKA activity in living cardiomyocytes using the FRET technique described in previous study [21]. In brief, β_1_β_2_AR-KO neonatal cardiomyocytes were infected with adenoviruses expressing β_2_AR or β_2_AR-C341A, along with cytoplasmic cAMP FRET biosensor ICUE3, plasma membrane targeted cAMP FRET biosensor PM-ICUE3, or PKA FRET biosensor AKAR2.2 [22], [23]. After overnight expression, cells were washed twice with PBS and challenged by 10 nM or 10 µM isoproterenol as indicated. Cells were treated with 2-bromopalmitic acid (100 µM, Sigma, MO) 15 minutes to inhibit palmitoylation of β_2_AR. Rolipram was added at final concentration of 10 µM to inhibit all PDE4 activities in the cells. Images were captured every 20 seconds and the ratio between emission of YFP and that of CFP was plotted as an indicator of dynamic activity change that was automatically analyzed by Metafluor software. Basal line substraction was performed using Origin software. Data analysis was performed by GraphPad Prism software.

### Statistical Analysis

All statistical analyses were performed using GraphPad Prism software. p<0.05 was considered statistically significant.

## Results

### Disruption of Palmitoylation of β_2_AR Enhances Agonist-induced cAMP-PKA Activation in the Cytoplasm and Myocyte Contraction Response

**Figure 1:**
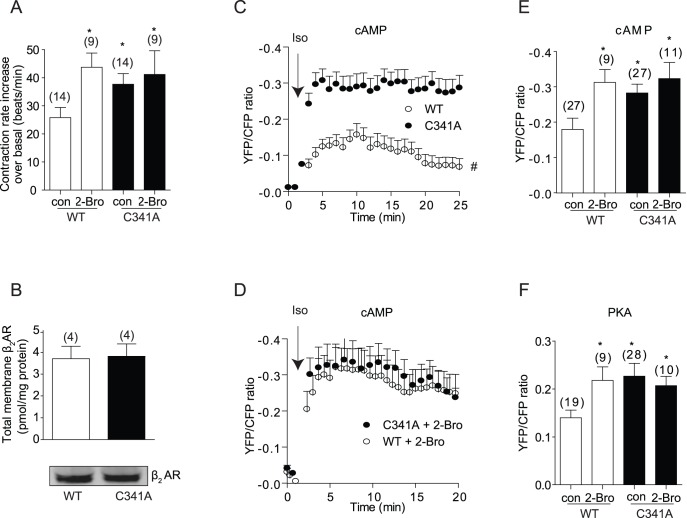
Stimulation of palmitoylation mutant β_2_AR leads to higher and sustained intracellular cAMP-PKA activities and higher increases in cardiomyocyte contraction rate. β_1_β_2_AR-KO cardiomyocytes expressing β_2_ARs via adenovirus infection were stimulated with isoproterenol (10 µM). The spontaneous contraction rate was recorded before and after isoproterenol stimulation. A) Quantification of isoproterenol-induced cardiomyocyte contraction rate increases over the baseline levels was plotted in the absence or presence of treatment with 2-bromopalmitic acid. B) 20 µg membrane protein containing β_2_ARs was determined by radioligand [^3^H] DHA binding to measure expression levels of β_2_AR and β_2_AR-C341A. The expressed receptors were also detected by Western blot using anti-flag antibody. β_1_β_2_AR-KO cardiomyocytes expressing β_2_ARs together with cytoplasmic cAMP FRET biosensor ICUE3 (C-E) or PKA activity biosensor AKAR 2.2 (F). (C) and (D) Cells were stimulated with isoproterenol (10 µM) to record the changes of FRET ratio. 2-Bro, 2-bromopalmitic acid (100 µM) was added 15 minutes before Iso stimulation; Quantification of isoproterenol-induced maximal increases in cAMP FRET ratio (E) and PKA FRET ratio (F) was plotted. In each panel, bar graphs were average from the maximal increases in the indicated number of cells (FRET assay) or dishes (beating assay) from at least three independent experiments. *, p<0.05 in comparison to WT control stimulated with Iso alone by one-way ANOVA; #, p<0.05 between WT and C341A stimulated with Iso by two-way ANOVA.

We used two complimentary strategies to examine the functional implication of palmitoylation of β_2_AR in cardiomyocytes. First, a point mutation was introduced on human β_2_AR to generate β_2_AR-C341A, in which the cysteine residue was replaced by alanine. Both mutant and wild type β_2_AR were expressed in cardiomyocytes lacking endogenous β_1_ and β_2_ARs (DKO). Activation of the mutant β_2_AR-C341A by 10 µM of isoproterenol induced a higher increase in myocyte contraction rate than wild type receptor (Figure 1A) even though both receptors displayed equivalent expression measured by [^3^H] dihydroalprenolol ligand binding and by Western blot (Figure 1B). Second, we applied 2-bromopalmitic acid to inhibit receptor palmitoylation [24], which selectively enhanced the contraction rate increase induced by wild type receptor, but not those by C341A mutant (Figure 1A). To validate the observation of functional contraction response, we examined the cAMP activities in the cytoplasm, which is relevant to PKA activation at the intracellular compartments such as sarcoplasmic reticulum, calcium storage for cellular calcium signaling and contractile response. We applied the cytoplasmic cAMP FRET sensor ICUE3 to measure the cAMP dynamics. Activation of β_2_AR-C341A induced higher cAMP FRET responses than WT receptor in the cytoplasm (Figure 1C and 1E). Inhibition of receptor palmitoylation with 2-bromopalmitic acid selectively enhanced the cAMP FRET signals in the cytoplasm induced by WT β_2_AR but not by β_2_AR-C341A, and abolished the difference between the two receptors (Figure 1D and 1E). These data are consistent with the cAMP accumulation measured by cAMP immunoassay in cell lysates (Figure S1). In agreement with cAMP data, activation of β_2_AR-C341A by isoproterenol induced higher increases in PKA activities measured by FRET biosensor AKAR2.2 in the cytoplasm than WT β_2_AR (Figure 1F). 2-bromopalmitic acid treatment selectively enhanced increases in PKA activities induced by stimulation of WT β_2_AR (Figure 1E).

### Disruption of Palmitoylation of β_2_AR Alters Spatio-temporal Distribution of Agonist-induced cAMP in Cardiomyocytes

**Figure 2:**
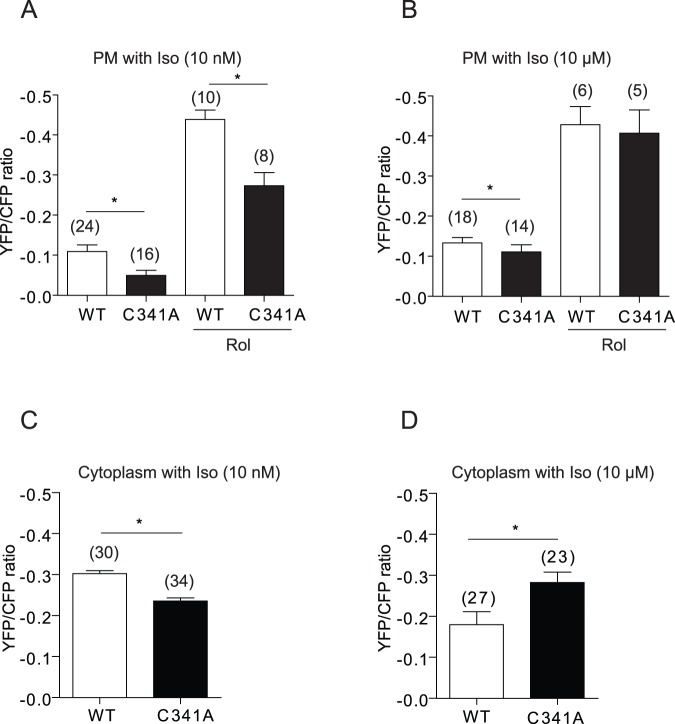
Mutation of palmitoylation reduces β_2_AR/G protein-dependent cAMP production in cardiomyocytes. β_1_β_2_AR-KO cardiomyocytes were infected with adenoviruses expressing β_2_ARs together with the plasma membrane-targeted cAMP FRET biosensor PM-ICUE3 (A and B) or cytoplasmic cAMP FRET biosensor ICUE3 (C and D). Cells were stimulated with 10 nM of isoproterenol (A and C) or 10 µM of isoproterenol (B and D). The maximal increases in FRET ratio were plotted. Rol, rolipram, was added together with Iso at 10 µM. Bar graphs were average from the maximal increases in time courses. *, p<0.05 by *t*-test in comparison to WT β_2_AR.

Previous study on β_2_AR palmitoylation in Chinese hamster fibroblasts suggests that mutation of the palmitoylation residue reduces β_2_AR/Gs coupling to activate adenylate cyclases for cAMP production on the isolated plasma membrane [14]. Here, we observed that intracellular cAMP and PKA levels in myocytes expressing β_2_AR-C341A were higher than those with WT receptor, leading higher contraction rate response under agonist stimulation (Figure 1). This observed difference suggests a potential alteration of subcellular distribution of cAMP signal between the plasma membrane and the cytoplasm under stimulation of the mutant β_2_AR-C341A. Besides Gs/AC-dependent cAMP production, the cAMP dynamics can also be modulated by β_2_AR-associated PDE4D for cAMP degradation [7], [17]. We envisioned that the mutant receptor also caused alteration in the membrane trafficking of β_2_AR and its associated PDE4D. To test this hypothesis, we first tried to validate the coupling efficiency between the mutant receptor and Gs/AC axis. We applied the plasma membrane-localized FRET biosensor to directly measure cAMP in the local domain produced by receptor activation in living myocytes. By using a low concentration of 10 nM isoproterenol, at which the ligand-bound receptor only activates Gs protein without undergoing internalization and Gi coupling [21], we selectively analyzed receptor/Gs coupling of the mutant β_2_AR-C341A. At minimal 10 nM isoproterenol, β_2_AR-C341A induced a lower cAMP FRET response at the plasma membrane than WT receptor (Figure 2A). Inhibition of PDE4 with rolipram enhanced the FRET responses induced by both receptors, but the difference between increases induced by two receptors still remained (Figure 2A). These data are consistent with previous publications showing that the mutant β_2_AR-C341A has impaired coupling to Gs protein for cAMP production [14]. At the same concentration of isoproterenol stimulation, β_2_AR-C341A induced a lower cAMP FRET response in the cytoplasm than WT receptor (Fig. 2C). However, at 10 µM isoproterenol stimulation, while the cAMP signals at the plasma membrane induced by C341A was lower than wild type receptor (Figure 2B), the cAMP signal in the cytoplasm induced by C341A was higher than wild type receptor (Figure 2D). We hypothesize that the trafficking of activated receptor and its associated PDE4D shape the subcellular cAMP signals at high concentration of isoproterenol stimulation. In supporting this notion, inhibition of PDE4 enhanced cAMP signals and normalized the difference between the two receptors in both measurements of cAMP in the cytoplasm by FRET assay and cAMP accumulation by immunoassay (Figure S2).

### Disruption of Palmitoylation of β_2_AR Reduces Receptor Association with PDE4D Isoforms at Both Resting and Stimulating Conditions

**Figure 3:**
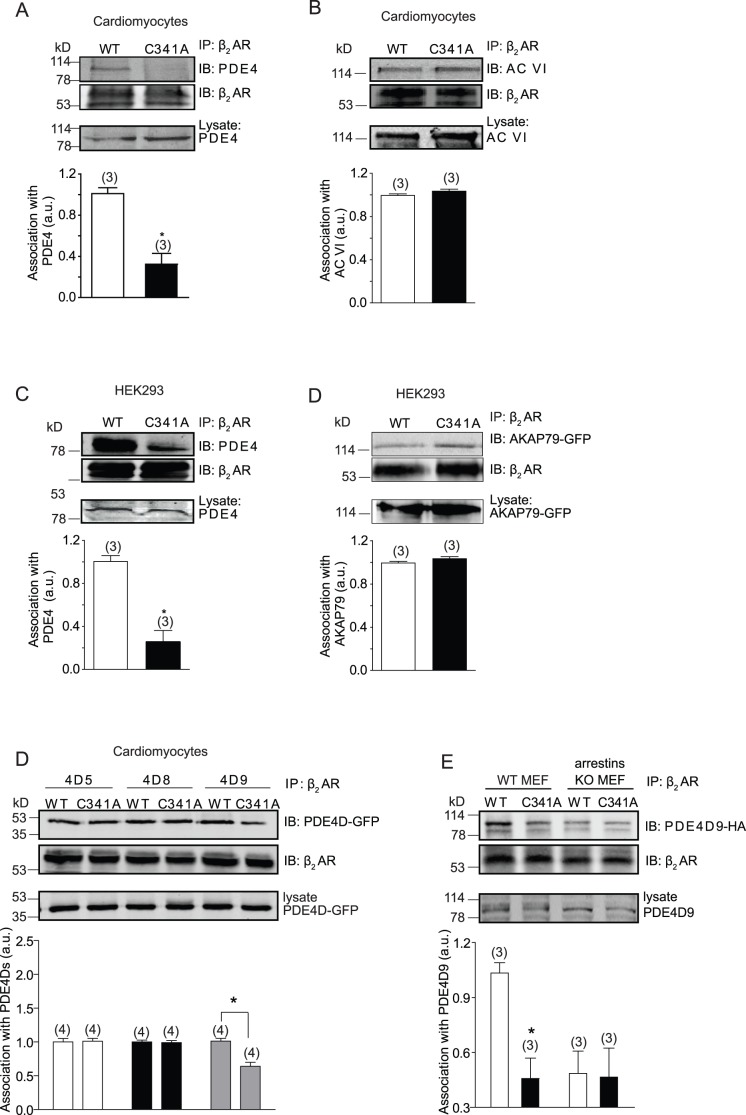
Palmitoylation is required for β_2_AR association with phosphodiesterase 4. Cells expressing flag-β_2_ARs alone (A and C) or together with adenylate cyclase VI (B), AKAP-GFP (D), GFP-PDE4D (E), or PDE4D9-HA (F) were lysed for immunoprecipitation with anti-flag M2 beads. The bound proteins were detected in Western blot by anti-flag, anti-GFP, anti-PDE4, anti-adenylate cyclase VI, anti-GFP, and anti-HA antibodies. Western blot was quantified and normalized against the IP protein. Bar graphs were average from 3–5 different experiments. *, p<0.05 by *t*-test between WT and C341A.

We then examined the association of PDE4D with mutant β_2_AR-C341A. β_2_AR-C341A displayed a reduced association with PDE4 in both HEK293 cells and neonatal cardiomyocytes (Figure 3A and 3C). In contrast, mutant β_2_AR-C341A did not change the receptor association with adenylate cyclase VI, one of the major adenylate cyclase isoforms expressed in cardiomyocytes as well as the receptor complex scaffold protein AKAP79 (Figure 3B and 3D). These data indicate that the mutation on β_2_AR palmitoylation site at C341 selectively reduces receptor association with PDE4 without globally disrupting association with AKAP and adenylate cyclase.

Among four PDE4 subfamilies, PDE4D subfamily contribute to 60% of total PDE4 enzymatic activities and regulate βAR signaling in cardiomyocytes [4]. Nine PDE4D isoforms have been identified which share identical catalytic domains and unique N terminal for distinct subcellular localization [25]. We have previously reported differential association of PDE4D enzymes with β_2_AR in cardiomyocytes at resting and stimulating conditions to modulate receptor-induced cAMP signal [7]. Only PDE4D9 displayed less association with β_2_AR-C341A at basal condition, consistent with the prominent role of this enzyme in controlling local cAMP and basal PKA phosphorylation of protein in receptor complex at resting state (Figure 3E and [7]). Consistently, overexpression dominant negative PDE4D9 or inhibition of PDE4 selectively enhanced cAMP levels in cells expressing wild type receptor, but not in those expressing β_2_AR-C341A at resting state (Figure S3).

**Figure 4:**
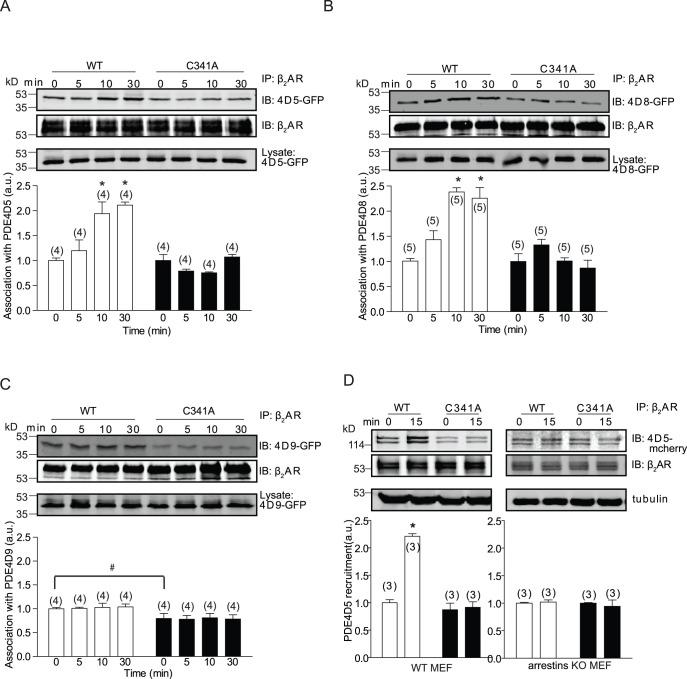
Palmitoylation of β_2_AR is required for agonist-dependent recruitment of arrestin-PDE4D complexes to the receptor. β_1_β_2_AR-KO cardiomyocytes expressing flag-β_2_ARs together with GFP-PDE4D5 (A), GFP-PDE4D8 (B), or GFP-PDE4D9 (C) were serum-starved for 30 minutes before stimulation with 10 µM of isoproterenol as indicated. Cell lysates were incubated with anti-flag M2 beads for co-immunoprecipitation of β_2_ARs and PDE4D proteins. The bound proteins were detected in Western blot by anti-flag and anti-GFP antibodies as indicated. D) Flag-β_2_ARs and PDE4D5-RFP were expressed in wild type and arrestins double knockout (KO) MEF cells. Co-IP was done like those in panel (A–C). The bound proteins were detected in Western blot by anti-flag and anti-RFP antibodies as indicated. Western blot was quantified and normalized against the IP protein. Bar graphs were average from 3–5 different experiments. *, p<0.05 by one-way ANOVA in comparison to WT β_2_AR without stimulation. ^#^, p<0.05 by *t*-test to WT control.

Arrestin plays a role in PDE4D association with β_2_AR in resting state [7]. We then investigated whether palmitoylation regulates the association among PDE4D isoforms, arrestins, and β_2_AR. Consistent with the data in cardiomyocytes, β_2_AR-C341A displayed less association with PDE4D9 than WT receptor at resting state in WT MEFs, but not in MEFs lacking arrestin proteins (Figure 3F), indicating a necessary role of arrestin in mediating palmitoylation-dependent binding of β_2_AR to PDE4D9. Upon agonist stimulation, PDE4D5 and PDE4D8, but not PDE4D9 are recruited to the activated receptor via scaffold protein β arrestin 2 (Figure 4A–4C and [7]). However, the recruitment of both PDE4D5 and 4D8 to β_2_AR-C341A was significantly reduced compared with that to WT β_2_AR (Figure 4A–4B), indicating the necessary role of palmitoylation for the β arrestin 2-dependent association of PDE4D with activated β_2_AR. Moreover, loss of β arrestin 2 abolished the agonist-induced recruitment of PDE4D5 to WT β_2_AR in MEFs (Figure 4D), further validating the critical role of β arrestin 2 in scaffolding PDE4Ds for regulating β_2_AR signaling.

**Figure 5:**
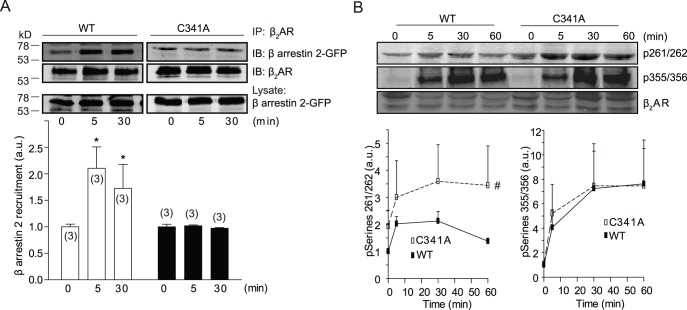
Palmitoylation of cysteine 341 of β_2_AR is required for agonist-induced recruitment of β arrestin 2, but not receptor phosphorylation by PKA and GRK. β_1_β_2_AR-KO cardiomyocytes expressing flag-β_2_ARs and β arrestin 2-GFP were serum-starved for 30 minutes before stimulation with 10 µM of isoproterenol as indicated. (A) Cell lysates were incubated with anti-flag M2 beads to immunoprecipitate flag-β_2_ARs. The bound proteins were detected in Western blot by anti-flag and anti-GFP antibodies. *, p<0.05 by *t*-test in comparison to the control without stimulation. (B) Cell lysates were used to detect receptor phosphorylation by PKA on serine 261/262 or GRKs on serine 355/356 by anti-phospho-serine 261/262 and anti-phospho-serine 355/356 specific antibodies as indicated. Western blots were quantified and normalized against the total β_2_AR expression. Bar graphs were average from 3–5 different experiments. ^#^, p<0.05 by two-way ANOVA in comparison to WT control.

### Disruption of Palmitoylation of β_2_AR Reduces Recruitment of Arrestin to the Phosphorylated Receptor Under Agonist Stimulation

**Figure 6:**
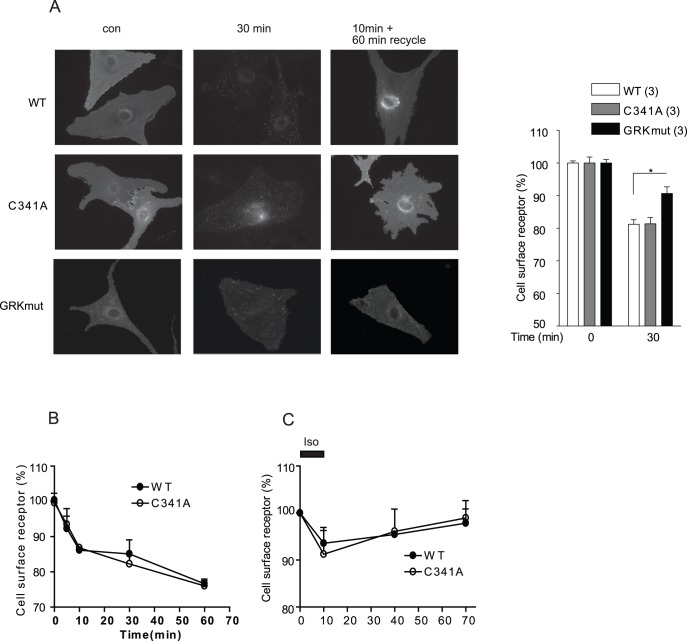
Mutation of palmitoylation site does not inhibit agonist-induced β_2_AR trafficking. A) β_1_β_2_AR-KO cardiomyocytes expressing wild type or mutant flag-β_2_ARs were stimulated with 10 µM isoproterenol to examine receptor internalization or followed by agonist removal to examine receptor recycling. Receptors were stained by anti-flag antibody M1 and Alex 488-conjugated goat-anti-mouse secondary antibody. Maximal internalization of different flag-β_2_ARs in β_1_β_2_AR-KO cardiomyocytes at 30 minute of stimulation was plotted in bar graph. Receptors remaining on the cell surface after internalization (B and D) or recycling (C) were quantified using Alex 488-conjugated flag antibody labeling. Quantitative measurements of cell surface receptor were average from at least three different experiments. *, p<0.05 by *t*-test in comparison to WT β_2_AR.

The agonist-induced binding of PDE4D to activated β_2_AR is dependent on the recruitment of arrestin. We suspected that the agonist-induced recruitment of β arrestin 2 to βAR is reduced by mutation of the palmitoylation site. In cardiomyocytes, β arrestin 2 had abundant expression whereas β arrestin 1 expression was low (Figure S4). β_2_AR-C341A displayed a reduced recruitment of β arrestin 2 upon agonist stimulation when compared to wild type receptor (Figure 5A). The recruitment of arrestin is dependent on receptor phosphorylation by GRK. Surprisingly, β_2_AR-C341A displayed normal phosphorylation by GRKs in comparison to the wild type β_2_AR upon isoproterenol stimulation. PKA phosphorylation of β_2_AR-C341A was higher at resting condition than wild type receptor; and the agonist-induced PKA phosphorylation was more sustained compared to that of WT β_2_AR (Figure 5B and Figure S5). This is consistent with previous report on higher PKA phosphorylation of mutant C341G receptor expressed in fibroblasts [16], [26]. Phosphorylation of β_2_AR plays a significant role in receptor desensitization through β arrestin-dependent pathway [8]. The observation of normal GRK phosphorylation but reduced recruitment of β arrestin 2 to β_2_AR-C341A (Figure 5A and 5B) prompted us to examine arrestin-dependent receptor internalization after agonist stimulation. To our surprise, β_2_AR-C341A displayed normal time-dependent internalization similar to that of WT receptor (Figure 6A and 6B). Moreover, both WT and C341A mutant displayed sufficient recycling upon isoproterenol removal (Figure 6A and 6C). Consequently, the cAMP signal induced by either wild type or mutant β_2_AR-C341A were sensitive to inhibition of Gi (Figure S6), indicating that both receptors are capable to couple to Gi after agonist-induced receptor translocation [27], [28]. As a control, a β_2_AR lacking critical GRK sites for arrestin binding and internalization showed impaired internalization (Figure 6A and 6D), consistent with early studies showing that β_2_AR internalization is dictated by GRK phosphorylation in cardiomyocytes [21].

### Disruption of Palmitoylation of β_2_AR Alters Agonist-induced Receptor Internalization Pathway

**Figure 7:**
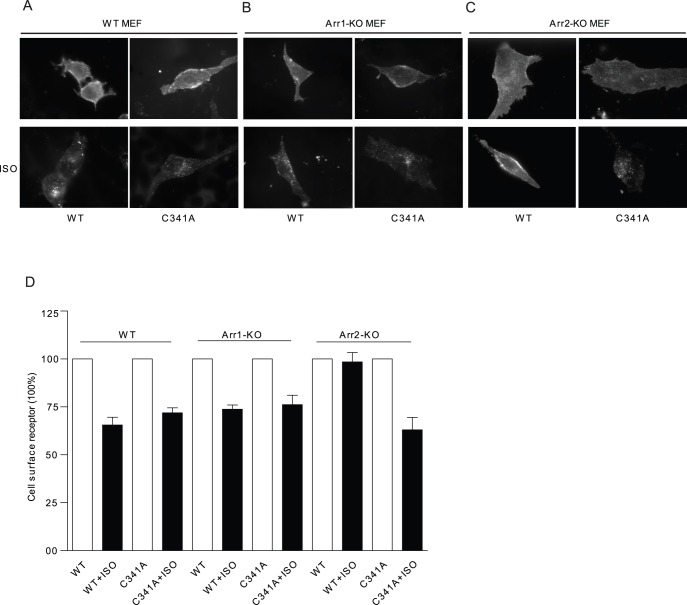
Mutation of palmitoylation site allows agonist-induced β_2_AR internalization via an arrestin-independent pathway. MEFs (WT, β arrestin 1-KO or β arrestin 2-KO) expressing flag-β_2_ARs or flag-β_2_AR-C341A were serum starved for 30 minutes before stimulation with isoproterenol 10 µM for 30 minutes to examine receptor internalization. A-C), cells were fixed, and receptors were stained by anti-flag antibody M1 and Alex 488-conjugated goat-anti-mouse secondary antibody. D), Cells surface receptor level after agonist stimulation was measured by FLISA method.

**Figure 8:**
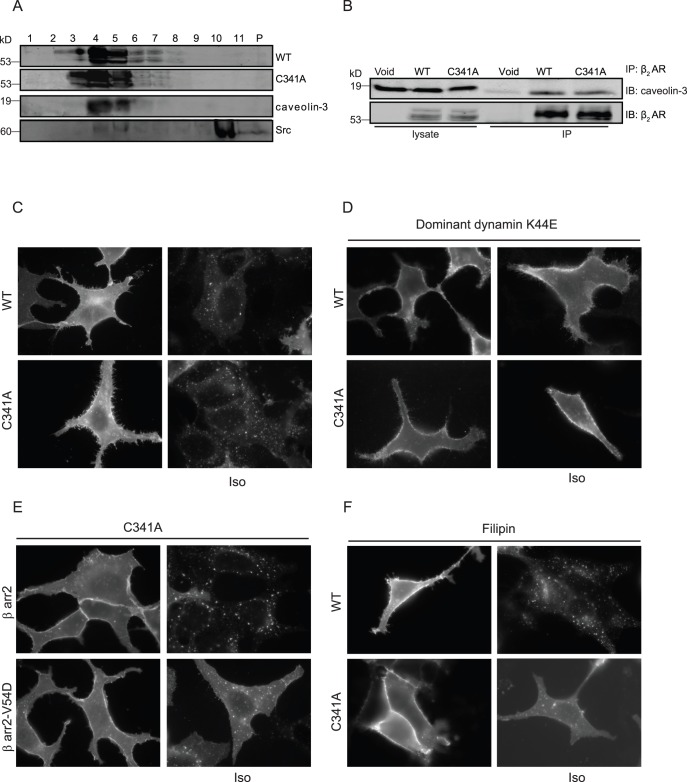
Mutation of palmitoylation site promotes caveolin-dependent β_2_AR internalization upon agonist stimulation. A) Membrane fractionations of β_1_β_2_AR-KO cardiomyocytes expressing β_2_AR or β_2_AR-C341A were blotted with caveolin-3, Src, and flag antibodies. P is the pellet fraction after centrifugation. B) Cell lysates were incubated with M2 beads for immunoprecipitation of flag-β_2_ARs to detect endogenous caveolin-3 association. (C–F) HEK293 cells expressing β_2_AR or together with other proteins were stimulated with 10 µM isoproterenol for 30 minutes to examine receptor internalization. C) Cells expressing flag-β_2_AR alone, D) cells expressing flag-β_2_ARs together dominant negative dynamin K44E, F) cell expressing flag-β_2_ARs were treated with 2 µg/ml filipin for 30 minutes, and E) cells expressing flag-β_2_AR-C341A together with β arrestin 2 (β arr2) or dominant negative β arrestin 2-V54D (β arr2-V54D), before stimulation with isoproterenol 10 µM for 30 minutes to examine receptor internalization. Cells were fixed, and receptors were stained by anti-flag antibody M1 and Alex 594-conjugated goat-anti-mouse secondary antibody.

We then further explored the mechanism underlying the agonist-induced internalization of β_2_AR-C341A with MEF cells lacking individual arrestin genes. Deletion of β arrestin 2, but not β arrestin 1 gene selectively blocked isoproterenol-induced internalization of WT receptor, but did not affect the internalization of β_2_AR-C341A (Figure 7). These data indicate that β_2_AR-C341A undergoes internalization via an arrestin-independent pathway. GPCR can undergo internalization via both arrestin- and caveolae-dependent pathways although both pathways are dependent on the dynamin GTPase [29], [30]. β_2_AR has been shown to be localized in caveolae/lipid rafts for proper signaling and function [31], we examined whether palmitoylation plays a role in β_2_AR localization in caveolae, thus modulating the receptor trafficking properties upon agonist stimulation. Both β_2_AR and β_2_AR-C341A were enriched in caveolae-rich membrane fraction, which was further supported by co-immunoprecipitation between receptor and caveolin-3 (Figure 8A and 8B). While β_2_AR and β_2_AR-C341A displayed normal internalization upon isoproterenol stimulation (Figure 8C), the receptor internalization was completely blocked by overexpression of dominant negative dynamin K44E in HEK293 cells (Figure 8D). Meanwhile, pretreatment with filipin, a drug that disrupts lipid rafts/caveolae [32], inhibited isoproterenol-induced internalization of β_2_AR-C341A (Figure 8F). In comparison, overexpression of dominant negative β arrestin 2 V54D only partially inhibited isoproterenol-induced internalization of β_2_AR-C341A (Figure 8E). Together, these data suggest that β_2_AR-C341A utilizes additional caveolin-dependent pathway for internalization that is dependent on dynamin GTPase.

## Discussion

Palmitoylation is a common feature shared by many G-protein coupled receptors. In the present study, we reveal a couple of new functional properties of palmitoylation of β_2_AR in function and signaling. First, palmitoylation of β_2_AR is required for receptor signaling complex formation. Disruption of palmitoylation of β_2_AR selectively abolishes the receptor interaction with β arrestin 2 and PDE4D enzymes for cAMP degradation without affecting the receptor association with AKAP and adenylate cyclase. Second, palmitoylation of β_2_AR modulate the receptor endocytic pathways under agonist stimulation. Mutation on palmitoylation site of β_2_AR shifts the receptor internalization from an arrestin-dependent pathway to a caveolae-dependent pathway. Thus, the mutant β_2_AR is able to undergo internalization without recruiting arrestin and PDE4 enzymes. This leads to altered redistribution of PDE4 enzymes from the plasma membrane to intracellular endosomal compartments, which changes the cAMP dynamics in these subcellular compartments under agonist stimulation of β_2_AR. As a result, activation of the mutant β_2_AR leads to higher intracellular cAMP level in cardiomyocytes to promote stronger PKA activity and myocyte contraction response.

Recent studies provide accumulating evidence on elaborated complex formation of GPCRs with their signaling partners. These data demonstrate a tightly regulated and dynamic equilibrium between the receptor complex with stimulatory adenylate cyclases for second messenger cAMP production and the receptor complex with inhibitory phosphodiesterases for cAMP hydrolysis. β_2_AR is known to bind to AKAP79 and AKAP250 [33], and both AKAP79 and mAKAP is shown to scaffold adenylate cyclases to facilitate signaling transduction efficiency and specificity [34]. On the contrary, β_2_AR binds to PDE4 enzymes in an arrestin-dependent manner [7], which shapes the spatiotemporal distribution of intracellular cAMP. In one recent example, relaxin receptor is able to form a stable complex with both AKAP79-anchored AC and arrestin-associated PDE4. This complex allows maintaining a tonic cAMP signal induced by the receptor [35]. We find that mutation of palmitoylation site on β_2_AR displays a reduced binding to PDE4D without affecting the binding to AKAP79 and AC. This reduced binding of PDE4D9 is due to that the mutant β_2_AR displays a reduced association of β arrestin 2 (Figure 3), similar to the effect of palmitoylation on the recruitment of arrestin to activated vasopressin receptor [36]. Under agonist stimulation, the mutant β_2_AR-C341A also displays reduced association with PDE4D5 and PDE4D8 (Figure 4), which is again due to a reduced recruitment of β arrestin 2 to activated receptor (Figure 5). Therefore, palmitoylation is required for β_2_AR binding to β arrestin 2, which is necessary to connect PDE4D enzymes to the receptor.

Despite the inability of the mutant β_2_AR-C341A to recruit β arrestin 2, the receptor is able to undergo agonist-induced internalization via a caveolae-dependent pathway. This is consistent with report that β_1_AR is able to internalize via both clathrin and caveolae-dependent pathways in HEK293 cells, which is dependent on receptor phosphorylation by GRK2 and PKA respectively [37]. Accordingly, the mutant β_2_AR-C341A displays normal GRK phosphorylation but an increased and prolonged PKA phosphorylation under agonist stimulation. The increased PKA phosphorylation may promote PKA-dependent internalization of the mutant receptor via the caveolae-dependent pathway. Meanwhile, mutation of GRK phosphorylation sites reduces agonist-induced internalization, in agreement with the critical role of GRK phosphorylation for arrestin-mediated β_2_AR internalization and desensitization [8]. Together, both GRK phosphorylation and palmitoylation are necessary for recruitment of arrestin for internalization of the activated β_2_AR; mutation of palmitoylation promotes PKA phosphorylation, and PKA-dependent internalization via caveolae-mediated pathway.

The structural properties involved in palmitoylation for selective binding of receptor to arrestin, but not to AKAPs remains to be determined. The mutant β_2_AR-C341 also displays normal association with caveolin, indicating that the modification is not required for caveolae-targeting. This observation is similar to those palmitoylation sites reported on caveolin-1, but different from palmitoylation-dependent targeting to lipid rafts by other proteins including G protein signaling proteins (RGS) [38], G proteins [39], SNAP proteins [40], and Ras proteins [41]. While the mutant β_2_AR-C341 undergoes internalization via the caveolae-dependent pathway, dominant negative dynamin K44E completely blocks the internalization (Figure 8F and 8D). The observations are consistent with other studies showing that GPCRs can undergo different internalization pathways including both arrestin-dependent and caveolin-dependent endocytosis [29], [30], and both internalization pathways require the activity of dynamin GTPase.

The β_2_AR-induced intracellular cAMP/PKA signaling is shaped by dynamic equilibrium between Gs/AC-mediated cAMP production and PDE-mediated cAMP hydrolysis. The mutant β_2_AR-C341A displays an impaired coupling to Gs protein for cAMP production. Here, the reduced association of PDE4D with mutant β_2_AR-C341A has profound effects on subcellular dynamics of cAMP in cardiac myocytes. The mutant β_2_AR-C341A undergoes internalization without recruiting the PDE4D, which alters the subcellular distribution of PDE4D and contributes to a higher and more sustained intracellular cAMP accumulation under agonist stimulation. Consequently, the mutant β_2_AR-C341A induces higher PKA activity and contraction response in cardiomyocytes. Together, we have shown that palmitoylation of β_2_AR modulates receptor signaling in cardiomyocytes via regulating the receptor-associated complexes. These complexes potentially limit the cAMP signaling in local environments, which not only contributes to receptor signaling specificity but also protects cells from overstimulation.

## Supporting Information

Figure S1 **β_1_β_2_AR-KO cardiomyocytes were infected with adenoviruses expressing flag-β_2_AR or flag-β_2_AR-C341A for 24 hours.** A) Total cAMP levels after 5 minutes Iso(10 µM) stimulation were measured using cAMP HTS immunoassay kit. 2-bromopalmitic acid (100 µM) was added 15 minutes before cells were lysed for cAMP assay. Bar graphs represent average from at least three experiments. *, p<0.05 in comparison to WT control stimulated with Iso alone by one-way ANOVA.
(EPS)

Figure S2 (A) β_1_β_2_AR-KO cardiomyocytes expressing β_2_ARs along with cAMP FRET biosensor ICUE3 were stimulated with 10 µM isoproterenol. Rolipram (Rol, 10 µM isoproterenol as indicated. The changes in ICUE3 FRET ratio were recorded, and the maximal changes in FRET ratio were plotted. (B) β_1_β_2_AR-KO cardiomyocytes expressing β_2_ARs were stimulated with 10 µM isoproterenol for 5 minutes. Rolipram was added together with isoproterenol as indicated. Cells were lysed and the cAMP accumulations were determined with using cAMP HTS immunoassay kit. Bar graphs represent average from at least three experiments. *, p<0.05 in comparison to WT control stimulated with Iso alone by one-way ANOVA.
(EPS)

Figure S3 **β_1_β_2_AR-KO cardiomyocytes expressing β_2_ARs along with dominant negative PDE4D9 as indicated were stimulated with 10 µM isoproterenol for 5 minutes.** Cells were lysed and the cAMP accumulations were determined with using cAMP HTS immunoassay kit. Bar graphs represent average from at least three experiments. *, p<0.05 in comparison to WT control stimulated with Iso alone by one-way ANOVA.
(EPS)

Figure S4 **Detection of endogenous β arrestin 1 and β arrestin 2 in cardiomyocytes by β arrestin 1 and β arrestin 2 antibodies.** Lane 1, cell lysate from HEK293 cells transiently expressing β arrestin 1-GFP; Lane 2, cell lysate from HEK293 cells transiently expressing β arrestin 2-GFP; Lane 3, cell lysate from neonatal cardiomyocytes.
(EPS)

Figure S5 **β_1_β_2_AR-KO cardiomyocytes expressing β_2_ARs were lysed for detection of PKA phosphorylation of serine 261/262 by anti-phospho-serine 261/262 specific antibody.** Western blots were quantified and normalized against the total β_2_AR expression. *, p<0.05 in comparison to WT control stimulated with Iso alone by *t*-test.
(EPS)

Figure S6 β_1_β_2_AR-KO cardiomyocytes transiently expressing β_2_AR (A) or β_2_AR-C341A (B) along with cAMP FRET biosensor ICUE3 were pretreated with PTX (300 ng/ml) for 2 hours before stimulation with 10 µM isoproterenol. The changes in cAMP FRET over baseline level were recorded. The maximal changes over the basal levels were plotted. *, p<0.05 in comparison to WT control stimulated with Iso alone by one-way ANOVA.
(EPS)
